# Supplementary material for: Urinary C5b-9 as a Prognostic Marker in IgA Nephropathy
Source: J Clin Med. 2022 Feb 3;11(3):820. doi: 10.3390/jcm11030820 (PMC8836759; doi:10.3390/jcm11030820)
Supplement: Supplementary file 1 [file jcm-11-00820-s001.zip › jcm-1547605-supplementary.pdf]

Supplementary Materials

Table S1. Comparison of baseline serum and corrected urinary C5b-9 levels according to Oxford classification.

Variable	Baseline Serum C5b-9 Levels (ng/mL)	*p*-Value	Baseline Corrected Urinary C5b-9 Levels	*p*-Value
Oxford classification				
M score 0	47.0 ± 36.3	0.797	287.2 ± 486.3	0.510
M score 1[1]	39.4 ± 27.2		279.5 ± 364.8	
E score 0	41.5 ± 35.6	0.471	275.1 ± 436.4	0.384
E score 1[2]	45.7 ± 26.5		301.9 ± 405.8	
S score 0	41.2 ± 33.1	>0.999	513.1 ± 551.4	0.012
S score 1[3]	44.1 ± 31.9		113.9 ± 156.4	
T score 0	37.9 ± 25.2	0.628	323.9 ± 434.5	0.191
T score 1, 2[4]	49.5 ± 38.3		201.9 ± 400.4	
C score 0	44.9 ± 32.7	0.586	306.9 ± 441.7	0.730
C score 1, 2[5]	32.1 ± 25.3		111.7 ± 164.5	

Data are presented as mean ± standard deviation for continuous variables and were analyzed by Mann-Whitney *U* test. [1] Mesangial hypercellularity score > 0.5; [2] Presence of endocapillary proliferation = present; [3] Segmental glomerulosclerosis/adhesion = present; [4] Severity of tubular atrophy/interstitial fibrosis (T1 = 26–50%; T2 > 50%); [5] Presence of crescent (C1 = 1–25%; C2 = 26–100%).

Table S2. Correlation between baseline and changes in serum and urinary C5b-9 levels 6 months after medical treatment according to the percentage of segmental sclerosis, global sclerosis, and their sum as a fraction of all glomeruli.

Variable	Global Sclerosis (%)	Segmental Sclerosis (%)	Global and Segmental Sclerosis (%)
Baseline serum C5b-9 levels (ng/mL)	r = −0.181<br>*p* = 0.421	r = −0.116<br>*p* = 0.607	r = −0.284<br>*p* = 0.201
Baseline corrected urinary C5b-9 levels	r = −0.242<br>*p* = 0.175	r = −0.400<br>*p* = 0.021	r = −0.387<br>*p* = 0.026
Changes in serum C5b-9 (%)	r = 0.219<br>*p* = 0.328	r = −0.148<br>*p* = 0.512	r = 0.123<br>*p* = 0.585
Changes in corrected urinary C5b-9 (%)	r = 0.057<br>*p* = 0.752	r = 0.216<br>*p* = 0.228	r = 0.131<br>*p* = 0.466

Data were analyzed by Spearman’s rank correlation coefficient.

Table S3. Comparison of changes in serum and urinary C5b-9 levels according to the Oxford classification.

Variable	Changes in Serum C5b-9 Levels (%)	*p*-Value	Changes in Corrected Urinary C5b-9 Levels (%)	*p*-Value
Oxford classification				
M score 0	59.5 ± 130.5	0.949	−72.0 ± 46.9	0.191
M score 1	82.7 ± 185.7		−19.8 ± 152.2	
E score 0	86.4 ± 156.0	0.471	−35.6 ± 133.2	0.603
E score 1	49.1 ± 165.2		−66.9 ± 57.0	
S score 0	151.8 ± 33.5	0.162	−70.9 ± 45.9	0.199
S score 1	33.5 ± 133.3		−26.1 ± 145.8	
T score 0	81.6 ± 163.5	0.974	−41.5 ± 135.3	0.638
T score 1, 2	58.6 ± 156.7		−52.3 ± 64.2	
C score 0	60.1 ± 135.9	>0.999	−38.7 ± 121.9	0.377
C score 1, 2	140.9 ± 290.1		−91.2 ± 10.2	

Data are presented as mean ± standard deviation for continuous variables and were analyzed by Mann-Whitney *U* test
